# Gratuitous Services Injurious When Given without Due Discrimination

**Published:** 1852-07

**Authors:** J. L. Levison


### ARTICLE X.

### *Gratuitous Services Injurious when given without due discrimination.*

In times like those we live in, where there exists a fearful competition, and when it is most true, "that the battle is not always to the strong," we affirm that evil, so far as the medical profession is concerned, is greatly aggravated by giving, indiscriminately, gratuitous services to all who apply for the same. A few of the evils may be enumerated. Whatever is given away, or, which may be had on mere application, is, in a general way, very little valued. Elemosynary assistance, like gratuitous medical or surgical advice, may have a demoralising tendency.

1st. Because the persons who are the recipients of charity, or of unpaid services, lose their own self-respect, and suffer corresponding degradation. That such is the case, compare persons so situated with those who use their own efforts to avoid being dependent on others. Further, that the degradation becomes greater by every fresh compromise. So that a moral scale might be drawn, indicating the frequency of these acts.

Men's minds when once they take a downward motion, are governed by the laws of *momentum* like natural bodies, and acquire an accelerated motion as they approach the earth.

Those, therefore, who trust to others on all occasions, ultimately acquire a crawling attitude, and then there will not be any disinclination to receive any thing that is offered ; or, any diffidence in asking for gratuitous services. We do not wish to be misunderstood—we are not speaking of the utterly destitute ; or, of those cases when some sudden and frightful accident occurs, requiring great care and constant attention, more than the means of the sufferer would enable him to procure : and hence, for such it is an advantage, that there are public hospitals and dispensaries. But it is our object to show, that those who now cheat the medical man of his fees—spend the money in sensual indulgences; and that if they were industrious and frugal, they will have ample means to pay for such important services. In their history, we find them preferring the beggar's life, by spending all they get on themselves, without bestowing any thought on the wants of the morrow.

My remarks apply, in particular, to a vast number who obtain the gratuitous service of men in private practice, such as general practitioners, aurists, occulists and dentists. It is not the very poor who alone avail themselves of getting cured without any gratuity. Tradesmen and others, who could, and ought to pay, seem to think that professional men are so ethereal, that they and their families can live without food!

Mere thanks are not a legal tender which can be exchanged with the baker, the butcher, the grocer, &c. And yet, we have known the families of such worthies, take the advantage of the *gratis* hours of professional men. And what is still more annoying, actually suppose, that they are conferring some supposed benefit, and in their vulgar tongue, say: "*Otherwise, Mr. ——— would not do it for nothing.*"

In my own practice, I could cite some shameful instances, the following sample will suffice:

One morning, a well dressed woman, having on a fine silk gown, fur cuffs and boa, a handsomely trimmed bonnet with a black veil, and a gold watch at her side, came to have some stumps extracted. On being shown into my surgery, she said: "This is your *gratis* morning!" Yes, mam, for the very poor.

*I wish you to take out my stumps, as your hour is not up yet?*

This coolness and impudence staggered me.

She sat down, and I soon removed the objects of her annoyance. But afterwards, I told her, she had done an injury to the very poor, as from that hour, in order to protect myself, I would not, in future, attend any one without a letter of recommendation.

Having taken one view of the subject, let us now turn to another, that there is one great advantage in attending the very poor in large towns, viz. That, in their cases, diseases are often seen in their extreme forms. For, it is worthy of remark, that the very poor have often their minds so saturated with care, or their habits are so reckless or dissolute, that they heed not the first symptoms of any affection. And it often happens, that both these classes of persons will bear the most frightful and painful maladies, until they are almost beyond endurance; and then they seek advice from those who devote some portion of their time to gratis services. And often, although such practitioners do not obtain the slightest pecuniary recompense, they have the advantage of becoming acquainted with the *phases* of long standing and malignant diseases.

In my own dental practice, I have for many years devoted some mornings to assist any suffering mortals, whether they paid or not; and I have had often to marvel at the evils of ignorance, and to perceive the real benefit of mental cultivation.

Much actual professional knowledge I have thus obtained, and many of the cases submitted to my treatment, often excited my deepest sympathy.

But I also learned the secret source, and influence of quacks, and their means of profit. When the poor put themselves to the inconvenience of paying, they the advertising, "Cheap Operator;" or, if a medical case, the victims of the charlatan is lured by promises of speedy cure, and low fees. Hence, those who desire to be so far independent, and still unwilling or unable to pay a guinea or half guinea fees, go to the cheap doctor. And, in both instances, the poor patient finds that he has, after all, the worse bargain.

I will relate an instance or two in my experience. My late esteemed friend, Richard Casson, Esq., an eminent surgeon, at Hull in Yorkshire, brought to me, on one occasion, a man with a very large swelling on his cheek. The patient declaring, that he had had it ever since Dr. ———, (a gunsmith and dentist,) had drawn his tooth! The abscess looked very inflamed and shining, and on pressing its sides, I felt some foreign body, which evidentally moved about. We then cross-examined the poor fellow, and he said, Dr. —— was drunk, and that he had torn with a claw, (as he used a huge sized key,) the inside of his cheek. It was the second molar on the right side of the upper jaw.

The patient said he had bled profusely, after the extraction of the tooth; so, in his fright, he had forgotten to ask for it. That he afterwards, applied for it, when the gunsmith-dentist swore that he had swallowed it.

There was a large cicatrix in the inner side of the cheek, through it I made an incision of rather more than an inch in length, which enabled me to remove a large carious tooth with three fangs. The man soon got better, and his health was ultimately restored.

I have another instance of the "cheap jack" operators. The poor fellow, having put himself under the treatment of a village blacksmith, who combined with his real craft, that of cow leech, horse doctor and dentist! It is in the latter department we have to speak of him.

He is represented as repudiating the art and mystery of dental or other science, "as stuff and nonsense," and brandishing a collossal key instrument in his giant hand, declared, that he could take any teeth away "with this ere instrument;" but he did not add, "*charges as in Paris,*" *for he only took six pence* for large teeth, and "little ones," he took out for three pence. I have in my own dental museum, a specimen of his handy work, and a demonstrable evidence of his prowess. It is a first molar and two bicuspids* of the upper jaw, with their *alveoli* and gums, which he (the blacksmith) had broken down, in his endeavor to remove the carious molar; but, which happening to be anchylosed, resisted all ordinary efforts of such an operator. However, the blacksmith had too much self-esteem to be beaten, so he applied, what may be literally called, "brute force," and the whole three had given way, unable to resist "the long and the strong pull" of his mighty

* In the English fairs, there are travellers, who sell, by what is called a Dutch auglurd, descending lower and lower, they are called "cheap jacket."

arm. This uncouth vulcan dentist, seemed more fitting to operate on some cyclops, rather than on a biped patient.

The specimen of clumsy surgery, was given by one of my brothers, as it had been presented to him by the surgeon, who attended the poor victim. For two years, portions of bone exfoliated, and extensive irritation was kept upon the system. And it cost the suffering man many pounds, and brought him the experience, when almost too late, that, at least, if in no other case, in dental and other surgical operations, "the cheapest is the dearest." J. L. Levison.

